# Frequency of pyrethroid resistance in human head louse treatment: systematic review and meta-analysis

**DOI:** 10.1051/parasite/2021083

**Published:** 2021-12-22

**Authors:** Jalal Mohammadi, Kourosh Azizi, Hamzeh Alipour, Mohsen Kalantari, Masoumeh Bagheri, Marzieh Shahriari-Namadi, Saeedeh Ebrahimi, Mohammad D. Moemenbellah-Fard

**Affiliations:** 1 Department of Biology and Control of Disease Vectors, School of Health, Shiraz University of Medical Sciences 71645 Shiraz Iran; 2 Research Center for Health Sciences, Institute of Health, Shiraz University of Medical Sciences 71645 Shiraz Iran

**Keywords:** Allele, Control, Ectoparasite, *Kdr* genes, *Pediculus*, Therapy, Pyrethrum

## Abstract

Head lice (*Pediculus humanus capitis*) are one of the most common insects causing infestations in humans worldwide, and infestation is associated with adverse socio-economic and public health effects. The development of genetic insensitivity (e.g., target site insensitivity = knockdown resistance or *kdr*) to topical insecticides has impaired effective treatment. Therefore, this study was undertaken to review and meta-analyze the frequency of pyrethroid resistance in treated head louse populations from the beginning of 2000 to the end of June 2021 worldwide. In order to accomplish this, all English language articles published over this period were extracted and reviewed. Statistical analyses of data were performed using fixed and random effect model tests in meta-analysis, Cochrane, meta-regression and I2 index. A total of 24 articles from an initial sample size of 5033 were accepted into this systematic review. The mean frequency of pyrethroid resistance was estimated to be 76.9%. In collected resistant lice, 64.4% were homozygote and 30.3% were heterozygote resistant. Globally, four countries (Australia, England, Israel, and Turkey) have 100% *kdr* gene frequencies, likely resulting in the ineffectiveness of pyrethrin- and pyrethroid-based pediculicides. The highest resistance recorded in these studies was against permethrin. This study shows that pyrethroid resistance is found at relatively high frequencies in many countries. As a result, treatment with current insecticides may not be effective and is likely the cause of increased levels of infestations. It is recommended that resistance status be evaluated prior to insecticide treatment, to increase efficacy.

## Abstract (fr)

Les poux de tête (*Pediculus humanus capitis*) sont l’un des insectes les plus courants à l’origine d’infestations chez l’homme dans le monde, et l’infestation est associée à des effets socio-économiques et de santé publique néfastes. Le développement d’une insensibilité génétique (par exemple, l’insensibilité au site cible = résistance knockdown ou *kdr*) aux insecticides topiques a altéré l’efficacité de leur traitement. Par conséquent, cette étude a été entreprise pour examiner et méta-analyser la fréquence de la résistance aux pyréthroïdes dans les populations de poux de tête étudiées du début 2000 à la fin juin 2021 dans le monde. Pour ce faire, tous les articles en anglais publiés au cours de cette période ont été extraits et examinés. Les analyses statistiques des données ont été effectuées à l’aide de tests de modèles à effets fixes et aléatoires dans la méta-analyse, Cochrane, méta-régression et indice I2. Un total de 24 articles provenant d’un échantillon initial de 5033 ont été acceptés dans cette revue systématique. La fréquence moyenne de la résistance aux pyréthroïdes a été estimée à 76,9 %. Chez les poux résistants collectés, 64,4 % étaient homozygotes résistants et 30,3 % étaient hétérozygotes résistants. À l’échelle mondiale, quatre pays (Australie, Angleterre, Israël et Turquie) ont des fréquences de gène *kdr* de 100 %, ce qui entraîne probablement une inefficacité des pédiculicides à base de pyréthrine et de pyréthrinoïde. La résistance la plus élevée enregistrée dans ces études était celle contre la perméthrine. Cette étude montre que la résistance aux pyréthroïdes est trouvée à des fréquences relativement élevées dans de nombreux pays. En conséquence, le traitement avec les insecticides actuels peut ne pas être efficace et est probablement la cause d’une augmentation des niveaux d’infestation. Il est recommandé d’évaluer le statut de résistance avant le traitement insecticide, pour augmenter son efficacité.

## Introduction

Infestations of humans by *Pediculus* lice (Pediculosis) are increasing both in developed and developing countries alike [6]. Humans host three different kinds of lice: head lice (*Pediculus humanus capitis* De Geer, 1767) (Anoplura: Pediculidae), body lice (*Pediculus humanus humanus*), and pubic lice (*Pthirus pubis*). Head lice usually live on the scalp and body lice are commonly found in the folds of clothing of infested people. Both feed exclusively on human blood.

The prevalence of head lice varies in different parts of the world, but it is higher in school children, adolescents and girls than in other groups [1, 44, 66]. Sporadic reports on the prevalence of head lice in school children show variable levels of pediculosis with the European Union reporting a 2.1% infestation rate [5], Greece 5.3% [60], Poland 16.3% [64], Ethiopia 65.7% [15], Syria 14.3% [35], and Iran 7.4–10.5% [52, 54]. The global prevalence rate of head louse infestation is about 19%, as reported in a recent meta-analysis-based systematic review [32]. These data reflect the scope and variability of this problem.

Treatment and control of pediculosis is of particular importance due to its widespread occurrence. Treatment is commonly performed using topical insecticides, including permethrin 1%, malathion 0.5%, lindane 1%, and oral ivermectin [67]. Initially, permethrin and lindane successfully treated 89.7% and 95% of cases of head lice infestations, respectively [36, 50]. Lindane is now widely banned due to neurological reasons and there are increasing levels of resistance to insecticides such as pyrethroids due to their extensive use in treating pediculosis [24, 34]. In recent decades, the efficacy of the most widely used pyrethroid, permethrin, for the treatment of pediculosis has decreased to an unacceptably low level in the United Kingdom [23].

Pyrethroids, such as permethrin, bind to voltage-sensitive sodium channels (VSSC) in the nervous system and cause prolonged opening of these channels. Rapid and uncontrolled sodium influx leads to nerve depolarization which eventually causes muscle paralysis and death [26, 29]. A common mechanism of insecticide resistance is target site insensitivity, such as knockdown resistance (*kdr*), where point mutations in the target site (VSSC) reduce the binding of insecticides (i.e., dichlorodiphenyl trichloro-ethane or DDT and permethrin), causing nerve insensitivity and resistance. Resistance to pyrethroids (and DDT) was first described in flying insects, such as houseflies *Musca domestica*, causing a sudden, sometimes reversible, “death like effect” and so-called knockdown resistance [58, 69].

This resistance is a heritable genetic trait caused by recessive allele mutations, which occur in a wide range of insects that have been exposed to either DDT or a pyrethroid, or both, at some point in their evolutionary history. *Kdr*-causing point mutations (e.g., M815I, T917I, and L920F) in the VSSC α-subunit gene have been identified in resistant lice and are used as markers of pyrethroid resistance [13, 25, 26]. Although the sole detection of *kdr* gene mutations may not directly predict clinical failure, their rising frequency in head louse populations coincides with publications on product failures in controlled studies [72].

In general, resistance to insecticides has led to failure to treat or incompletely treat pediculosis, increasing its varied prevalence and intensity worldwide during the last three decades. As a result, it is necessary to use alternative insecticides or other treatment approaches. Preliminary knowledge of the frequency of genetic resistance in human head lice to topical insecticides is of particular importance in order to determine the use of appropriate treatment protocols [19, 24]. Therefore, the present study aimed to investigate the frequency of pyrethroid resistance to pediculicides via a systematic review and meta-analysis.

## Methods

A systematic review and meta-analysis were conducted to investigate pyrethroid resistance in head lice and its treatment. The Preferred Reporting Items for Systematic Reviews and Meta-Analyses (PRISMA) standard guideline was used to follow up the review process and report findings [45].

### Search strategy and selection criteria

This review focused on studies about *kdr* gene mutations and treatment of head lice that were published in English language journals between the year 2000 and June 2021. The Scopus, Web of Science, PubMed (including Medline), Cochrane database library and Science Direct databases were searched in medical subject headings (MeSH) and relevant keywords: Resistance, Knockdown Resistance, Insecticide Resistance, Pyrethroid Resistance, Pediculicide Resistance, Genetic Diversity, Molecular Monitoring, Resistance Mutations, Head Lice, Head Louse, Pediculosis, and Treatment. They were used in isolation or combination through the Boolean method.

### Inclusion and exclusion criteria

All English-language articles published worldwide on *kdr* gene mutations and human head lice treatment, which were of high quality, were entered into the study. Articles of low quality as outlined in the next paragraph, studies conducted on insects other than lice, uncertainty of mutation, and no reporting of resistance frequency were excluded from the study. Additionally, review studies, meta-analyses, case reports or series of cases were excluded.

### Quality assessment

The quality of the articles was assessed using the Strobe checklist (Strengthening the Reporting of Observational Studies in Epidemiology) [68]. This checklist has 22 parts that were scored based on the importance of each section, the lowest score of this checklist was 15 and the maximum was 33. In this study, an acceptable score of 20 was considered [16].

### Screening and data extraction

The search results were imported into Endnote software v.x8-1 and duplicate titles were deleted. Selected studies were entered into abstract reading and were checked against the inclusion criteria. Of these, the relevant studies were selected for independent full-text reading by two researchers and a third person as the expert-epidemiologist checked the results. Reasons for the rejection of studies were mentioned and in case of disagreement between the researchers, the perspective of a third researcher was sought. A checklist was used to extract data from the selected studies in terms of the sample size, study location, study period, type of study, pyrethroid resistance, type of mutation of *kdr* genes, and type of treatment.

### Selection of articles

**Figure 1:**
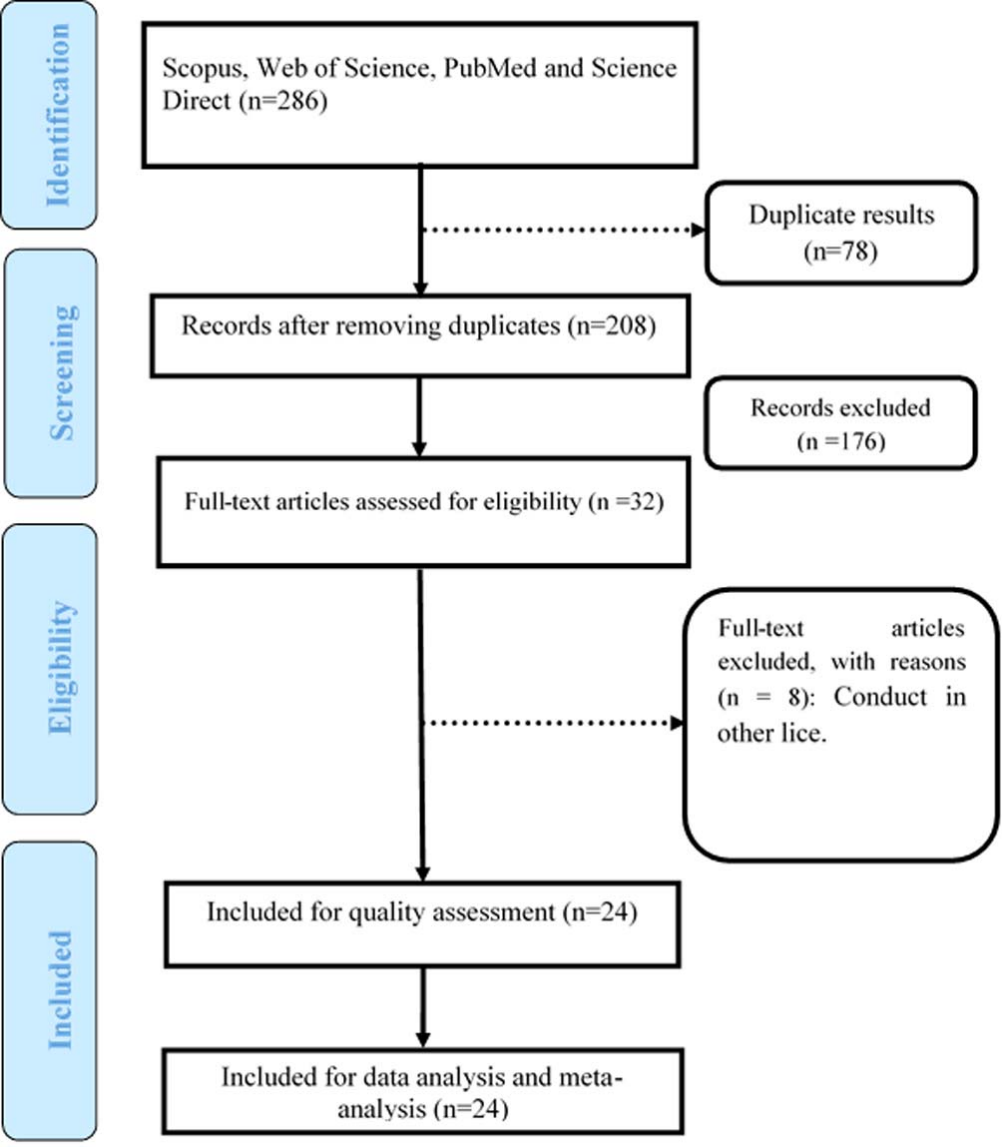
The PRISMA flow chart diagram.

By searching databases, 286 studies were extracted. Initially, the articles were entered into Endnote software and after an initial review, 78 articles were removed from the study due to duplication. Then, by reviewing the titles and abstracts of articles, 176 articles were removed due to irrelevance and after reviewing the full text of articles, 8 articles were excluded due to investigation of other lice species. Finally, 24 articles met the inclusion and eligibility criteria and entered the process of systematic review (Fig. 1).

## Results

**Figure 2:**
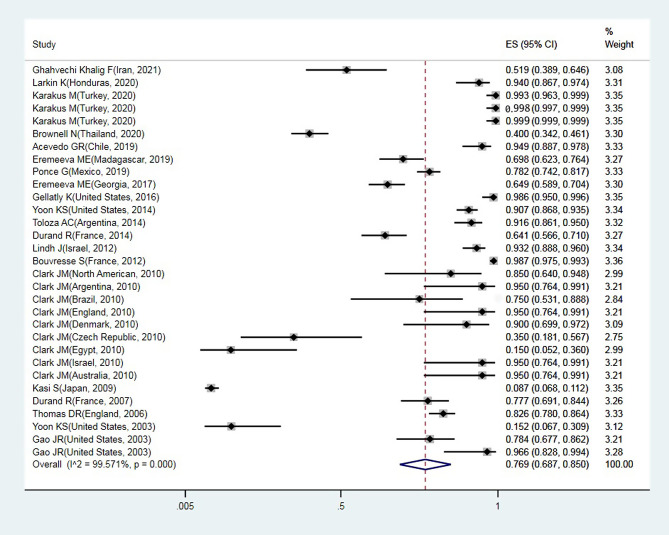
Forest plots of the proportion of resistance in lice and 95% confidence interval based on a random effect model in meta-analysis.

**Figure 3:**
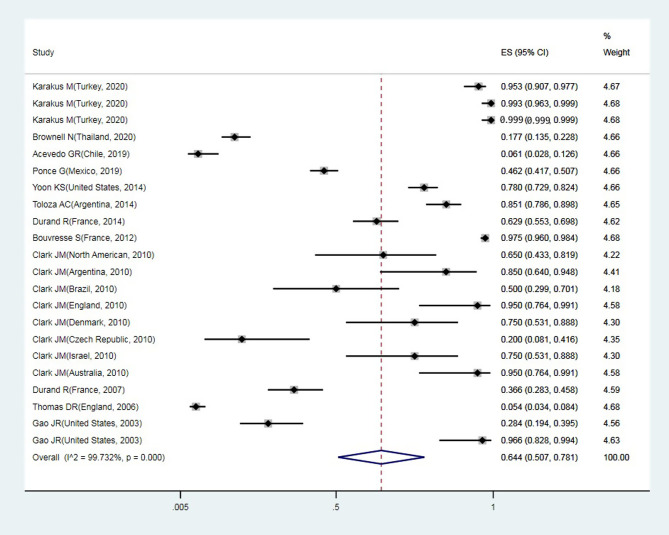
Forest plots of the proportion of homozygote resistant and 95% confidence interval based on a random effect model in meta-analysis.

**Figure 4:**
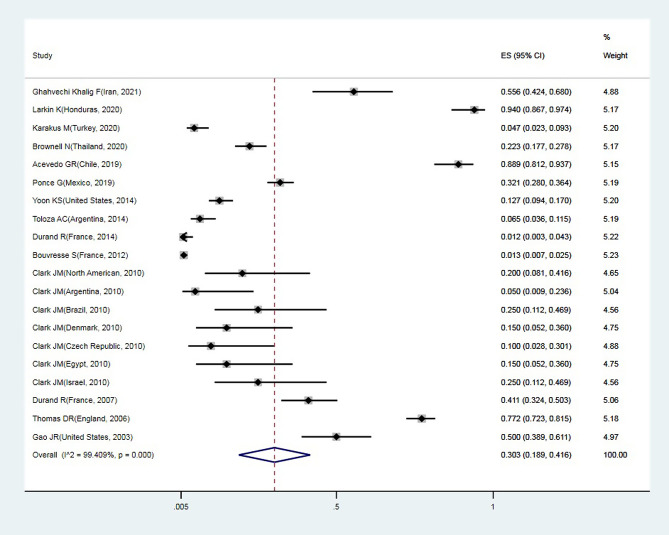
Forest plots of the proportion of heterozygote resistance and 95% confidence interval based on a random effect model in meta-analysis.

**Figure 5:**
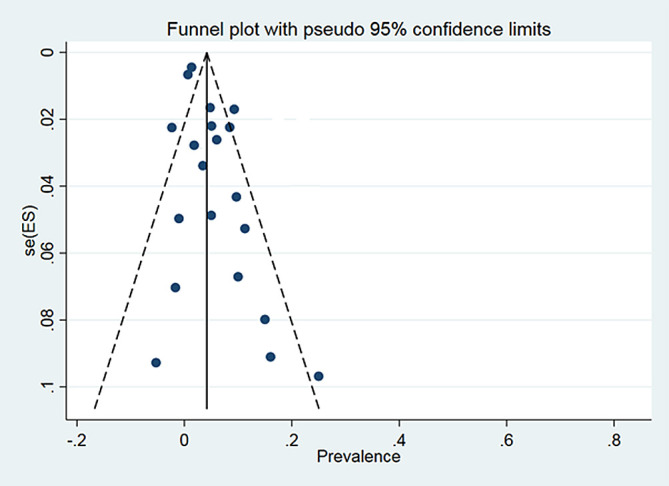
Funnel chart of proportion resistance in the selected studies.

**Figure 6:**
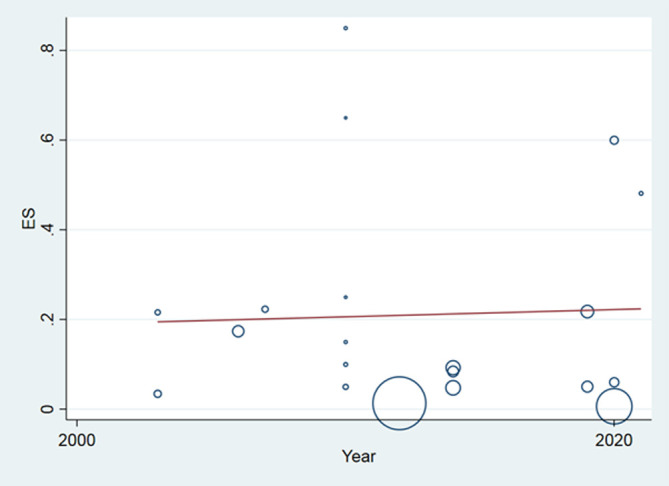
Meta regression chart of the proportion of resistance on the study year.

A total of 24 articles from an initial sample size of 5033 that were conducted between 2000 and 2021 were included in this study. The characteristics of the surveyed studies are presented in Table 1. Based on these findings, the globally reported mean frequency of pyrethroid resistance was estimated to be 76.9% (95%, CI: 68.7–85). In collected lice populations with *kdr* mutations, 64.4% were homozygote resistant and 30.3% heterozygote resistant (Figs. 2–4). The publication bias was investigated using a funnel plot, and due to the symmetry of the diagram, it can be assumed that diffusion bias did not occur, and the Egger test also confirmed it (*p* = 0.032) (Fig. 5). The relationship between study year and resistance status revealed that with increasing study year the frequency of resistance also increased (Fig. 6).

**Table 1:** General characteristics of the included studied in the data analysis process.

Authors	Year of study	Place of study	Sample size	*Kdr* allele mutation	Proportion *kdr* (%)
[30]	2021	Iran	54	K794E, F815I, and N818D	51
[41]	2020	Honduras	83	T917I	93.9
[37]	2020	Turkey	150	T917I	100
				L920F	99.3
				M815I	100
[8]	2020	Thailand	260	T917I	40
[59]	2019	Chile	99	T917I	94.9
[22]	2019	Madagascar	159	T917I	70
[57]	2019	Mexico	468	T929I	78.2
[21]	2017	Georgia	259	T917I	64.86
[29]	2016	United States	141	M815I, T917I, and L920F	98.4
[72]	2014	United States	291	T917I	90.7
[62]	2014	Argentina	154	M815I, T917I, and L920F	91.6
[18]	2014	France	167	M815I, T917I, and L920F	64.1
[46]	2012	Israel	192	M815I, T929I and I932F	93
[7]	2012	France	670	T917I and L920F	98.7
[13]	2010	North America	20	M815I, T917I and L920F	85.25
		Argentina			93
		Brazil			75
		England			100
		Denmark			92
		Czech Republic			33
		Egypt			15
		Israel			100
		South Korea			0
		Thailand			0
		New Guinea			0
		Australia			100
[38]	2009	Japan	630	M815I, T929I, and L932F	8.7
[14]	2008	Argentina	120	–	–
[20]	2007	France	112	T929I	77.8
[61]	2006	England	316	T917I	82.6
[40]	2006	Denmark	208	T929I and L932F	–
[70]	2003	United States	33	T929I and L932F	15
[28]	2003	United States	74	T929I and L932F	78
			29		97
[71]	2004	United States	121	T929I and L932F	-
[43]	2000	England	223	T929I and L932F	-

From studies conducted in 21 countries as outlined in 24 screened articles, the head louse sample populations collected from four countries (Australia, England, Israel, and Turkey) have complete (100%) *kdr* allele frequencies, suggesting that pyrethrin- and pyrethroid-based pediculicides are ineffective in these areas. The rest of them (17) reported frequencies ranging from zero to 99.3%, pointing to incomplete allele phenotypes. The largest (670) and the lowest (20) head lice sample sizes were attributed to studies conducted by French and American researchers, respectively.

A total of 40 *kdr* allele mutations were discovered in these screened articles, of which the first (30%) and the second (20%) most frequently identified allele phenotypes were T917I and T932I (amino acid Threonine replaced by Isoleucine at loci number: 917 and 932), respectively. So, half (50%) of all reported mutations have so far been attributed to TI conversion.

## Discussion

Based on the present findings, more than 70% of sampled human head lice were resistant to pyrethroid insecticides, and this rate has been increasing in recent decades, possibly following enhanced surveillance for pediculosis from head lice. Consequently, it is recommended that one should first determine the *kdr* allele frequencies in local human head louse populations, outline its zygote status and gene mutation type, design effective treatment methods, and then treat patients.

One possible reason for the detection of different levels of pyrethroid resistance in different parts of the world is the fact that different methods were used to diagnose genetic resistance in local head louse populations. Another factor could be the discrepancies between head louse lineages (see clades below) over different continents. Most probably, frequent application of various organochlorine and/or pyrethroid insecticides, and hence the resulting selection pressure against head lice could have given rise to the different levels of *kdr* allele frequencies in different regions allowing only heterozygote first to survive and reproduce. Different populations in terms of age and gender over disparate seasons could also be involved.

As mentioned above, pyrethroid target receptor mutation is a heritable recessive trait caused by persistent exposure to the above-named insecticides. Most often, refractoriness to pyrethrin- and pyrethroid-based pediculicides (and less to the organochlorine DDT) is caused by *kdr*-type mutations in the VSSC *α* – subunit gene of head lice. The head louse sample populations collected from five countries (Turkey, Australia, England, Israel, and Uruguay) were found to have complete (100%) *kdr* allele frequencies, suggesting that pyrethrin- and pyrethroid-based pediculicides are ineffective and their applications should be stopped in these areas [33].

The findings of this meta-analysis on *kdr* gene frequencies revealed that about 33% of human head lice were sensitive to the currently used insecticides, such as permethrin, in different parts of the world. However, most of them (≈67%) were resistant to the treatments. From our analysis, it is evident that most (81%) countries in the review were still in the incomplete phase for the selection of *kdr* allele mutations. This finding indicates an increasing risk associated with the extensive and mismanaged use of over-the-counter (OTC) pediculicides, such as permethrin, in mostly developed parts of the world. TI amino acid conversion constituted 50% of the identified phenotypes, which may substantiate the higher genetic plasticity of pyrethroid resistance due to this phenotypic mutation with respect to other types of mutations recorded so far.

To serve as an example of the variable sensitivity of head louse populations to different pediculicides, 10 years of research in North America revealed that the frequency of pyrethroid resistance between 1999 and 2009 was about 84.4%. However, this frequency was reported to be 97.1% in 2008 and 99.6% between 2007 and 2009. This finding demonstrates that the frequency of resistance increased in those years in this region [72]. These authors had earlier observed permethrin resistance in human head lice in California and Florida, USA but they were susceptible to the long-prohibited organochlorine lindane [71]. In susceptible populations of head louse, these recessive resistance alleles seem to be scarce. It is thus postulated that increased frequency of diagnosis and treatment would cause stronger selection pressure for pediculicide insensitivity, paving the way for heterozygotes to spread first, while reversion of head louse target receptor coding genes to susceptible allele status against a specific pediculicide formulation could emanate from its lack of application during the evolutionary history.

Various techniques have been used to diagnose genetic resistance. Polymerase chain reaction (PCR) was used to screen for mutations in T917I, L920F, M815I alleles, and detect *kdr* mutation in selected head louse populations in Turkey [37]. This method required small amounts of DNA for analysis, which could be extracted from lice, it could be carried out in a simple laboratory environment, and was suitable for *kdr* allele mutation screening [21, 26]. Quantitative sequencing (QS) for screening mutations causing the T917I, M815I and L932F amino acid conversions has also been implemented in head lice. Due to its speed, accuracy and simplicity, this method was a good candidate for screening resistant lice on a large scale [13, 42]. However, studies have shown that this method was mostly used to monitor and survey the levels of high frequencies of genetic mutations in lice populations [12, 29]. Real-time PCR (rtPASA) is another method used to monitor mutations based on frequency change, which has been used for low frequency [13]. Use of the serial invasive signal amplification reaction (SISAR) protocol to screen and diagnose *kdr* mutations was another development in this field [39]. This method was applied to identify single nucleotide polymorphisms and was an effective method for detecting heterozygous genetic mutations and Hardy–Weinberg equilibrium in lice [29, 47]. The use of this method for *kdr* alleles screening was also recommended in the United States and Canada [12]. In general, based on the findings of the present study and other studies, screening to diagnose pyrethroid resistance using simple, inexpensive and rapid laboratory methods is essential in order to select an appropriate treatment for the control of pediculosis.

Pediculosis caused by head lice is a major public health concern due to increased frequency and operations to contain these parasites pose even higher risks to human populations than the infestation itself [56]. A range of intervention methods are available for the control of head lice. The mainstay of therapy has overtly been the use of insecticides [52]. Treatment with pyrethroid insecticides resulted in a high degree of resistance, even though insensitivities to lindane had also been reported. As a result, it is necessary to use effective drugs with different active ingredients to treat the infestation. Some researchers have resorted to the use of “green” formulations [11, 51] including the oil of the eucalyptus plant species [63], lavender plus peppermint [4], tea tree oil [17], and extracts of citrus for the treatment of pediculosis [27]. Others have recommended the use of physically active dimeticones to treat head lice infestation [19]. Dimeticone lotion causes suffocation and should be applied to the scalp twice for 8 h, but its gel needed only 15 min to eliminate head lice [9]. Other recommended treatments for pediculosis included Crotamiton 10%, oral ivermectin, benzyl alcohol 5%, and Spinosad 0.9% [48, 49]. Isopropyl myristate dissolves the surface wax of lice, which leads to dehydration and death of lice [10]. Desiccation can be performed using heat-generating devices such as Louse Buster, which leads to water loss and death followed by manual removal using a comb [31, 53, 55].

Lice species are subdivided into clade haplotypes in terms of genome and geographical location. High and rapid diversification into different phylogenetic clades indicates the association between humanoids and head lice dating back to millions of years ago [3]. Head lice have thus globally been differentiated into six clades based on their mitochondrial DNA data. These haplotypes are named into clades A–F. These are grouped according to their territorial propagation. There is, therefore, an essential need to monitor these mutations through geographically specific genetic biomarkers [26] because of increasing failures to first-line treatment since allele mutations related to pyrethroid resistance differ between regions. There is clade A worldwide, clade B mainly in the United States, Europe and Australia, clade C in Africa and Asia, clade D in sub-Saharan Africa, clade E in West Africa, and clade F in Argentina and Mexico [2]. Among the above clades, clade C has more genetic diversity [6, 65]. International travel has caused the spread of clades to other regions and genetic interaction between them, especially clades A and C [26, 65]. It is speculated that genetic exchange between them is rapidly carried out due to the short life cycle of lice and the constant proximity of different types of clades. High and effective genetic exchange and diversity can lead to the spread of resistance genes, but on the contrary also to the maintenance of a reservoir of susceptibility. There is no available data on this topic regarding the dynamics of resistance.

In general, according to the findings of the present study, resistance to permethrin has increased worldwide due to *kdr* allelic mutations selected by the frequent use of topical pyrethroid and permethrin formulations. Accordingly, it is necessary to use alternative strategies and effective treatment approaches.

## Limitations

Limitations of this study included confinement to English language-based papers. No papers investigated the prevalence of all type *kdr* resistant alleles. Studies were in different years and different countries in which therapeutic approaches may have differed. There was also much heterogeneity between these studies.

## Conclusions

For the first time, this systematic review and meta-analysis attempted to examine, and found a relatively high frequency of pyrethroid resistance among human head louse populations in different countries. It could be concluded that there has been no study on the dynamics of resistance in human head lice so far. As a result, treatment with current pyrethroids and pyrethrins may not be efficacious in many cases. Based on this, it is recommended that drug resistance be evaluated first and then treatment be initiated with appropriate and effective protocols.

## Conflict of interests

None is declared by the authors who have no competing financial interests or personal relationships that could have appeared to influence this review paper.
